# Breastfeeding-friendly policies and programs in three public Universities in Ghana

**DOI:** 10.1186/s13006-022-00468-7

**Published:** 2022-04-11

**Authors:** Fred Yao Gbagbo, Jacqueline Nkrumah

**Affiliations:** Faculty of Science Education, Department of Health Administration and Education, Central Region, University of Education, P.O Box 25, Winneba, West African Ghana

**Keywords:** Breastfeeding, Ghana, Policies, Programs, Sustainable-Development-Goals, Universities

## Abstract

**Background:**

The United Nations through its Sustainable Development Goals (SDG) 3 and 5 has championed Women empowerment for exclusive breastfeeding through various action plans and expected the concept to be decentralized through locally mandatory implementation of various institutional policies and programs in member Countries. Using Kabeer’s empowerment concept, the authors in this paper assessed availability and implementation of breastfeeding policies and programs in three public universities in Ghana.

**Methods:**

The study design was an exploratory-descriptive-case study involving university employees and student mothers from three public universities in Ghana. The universities were selected via simple random approach whilst selection of participants was purposive. Data were collected between April and July 2018 using an unstructured interview guide developed by the authors, audio recordings, field notes and desktop review of documents. Manual thematic analysis of data was done to present results descriptively. The University of Cape-Coast Ethics Review Board approved the study.

**Results:**

Thirty-six respondents participated in the study. Three main themes (Breastfeeding policy and programs, Institutional support, and views on Breastfeeding/Childcare support) emerged. Despite being gender/child friendly, none of the universities in this study has a formal breastfeeding/childcare policy/program and there are no immediate policy plans for on-campus facilities to enhance breastfeeding. Financial cost emerged as a major challenge hindering the universities from implementing a policy/program in this regard. On the part of student mothers, lack of legal protection, lack of breastfeeding-friendly university policies, inadequate availability of breastfeeding facilities, and insufficient awareness of the importance of breastfeeding among nursing mothers has been a major setback for breastfeeding on campus, hence nursing mothers continue to make personal but challenging arrangements for breastfeeding on university campuses.

**Conclusions:**

The study findings reflect negative implications for childcare as it affects optimal child nutrition, hence impacting on achieving the SDGs 3 and 5 in Ghana. The authors recommend introducing formal breastfeeding-friendly policies/programs as one of the criteria for accreditation of universities in Ghana to enhance optimal childcare and sound maternal mind for studies and/or work once there is an assurance of child safety and proximity to breastfeed on demand.

## Background

Women’s empowerment has remained a focal part of the United Nations (UN) declarations on women’s issues since 1975 because of its importance in global development [1]. In various international development frameworks such as the UN Sustainable Development Goals 3 and 5; the UN conventions on child care/rights, women empowerment is considered an intrinsic goal for national development because of its pivotal roles in child health, care, growth and development [2–4]. These initiatives have stimulated women’s increasing engagement in various social spheres of life across diverse settings as women are increasingly bridging huge gender gaps with men in various social domains [5]. Gender gaps in basic, secondary, and tertiary education have shrunk substantially in many countries over the past three decades [6]. These improvements in the educational status of women are paving way for more women to participate in politics, governance, formal labor market, and public life [7–10].

The positive outcomes of women's empowerment and development are evident in Ghana [11]. Presently, Ghana boasts of increased female enrollment at all levels of education and formal employment. Female gross enrollment in second and tertiary institutions stood at about 60% and 13% respectively in 2015 compared to 34% and less than 1% respectively in 1991 [12]. Even though the proportion of women in government is quite low, the percentage of women engaged in economic activities is high in Ghana’s formal sector [13]. The entry of more women of childbearing age into job markets and tertiary institutions have made it increasingly imperative that support for breastfeeding and childcare are provided at workplaces and academic institutions. This study adopted an existing concept of empowerment [14] and explored critical roles played in promoting the development and rights of women in paid employment and educational opportunities linking it to the support offered to students and employee mothers in public universities in Ghana. The adopted concept [14] explained women empowerment as a process by which women take control and ownership of their lives through expansion of their choices. In furtherance to this, it has been postulated that the concept of women's empowerment focuses on the capability of women for self-determination by taking control over their own circumstances and to realize their aspirations in order to live a life they have reason to value.

[14, 15]. In this regard, the emphasis here is on ‘agency’ which is described as the ability to define goals, have meaningful choices, and to act to achieve desired outcomes [14].

Ghana has many legal and institutional frameworks supporting women's empowerment. For instance, article 17(1) and (2) of the 1992 constitution guarantee gender equality. Apart from the constitution, other frameworks available for promoting women's empowerment are: 1) Executive Instrument 8 of 2001 which established the Ministry of Gender, Children and Social Protection (MoGCSP) [16], 2) The various Growth and Poverty Reduction Strategies (i.e. World Bank and Ghana's poverty reduction strategies) [17] 3) Achieving the Free Compulsory Universal Primary Education [18] and the Free Senior High School Education policy as appropriate replacement to the Progressive Free Senior High Policy [19]. In terms of pressure from civil society, institutional actors, such as bilateral agencies and non-governmental organizations also influence policy and discuss issues related to women’s rights and continued empowerment [20].

Ghana being a member of UN and affiliate organizations like the International Labor Organization (ILO), has responsibilities to conventions and recommendations of UN affiliates. Yet, apart from the Labor Act (Act 651of 2003) which guarantee twelve weeks paid maternity leave and a further extension in the case of multiple births or intrapartum and postpartum complications, and the Breastfeeding Promotion Regulation Act of 2000 (LI 667), regulating the marketing of breastmilk substitute, it appears Ghana has not fully implemented the ILO’s conventions (No. 103 of 1952; No. 3, 1999; No, 156 of 1981; No. 183, 2000) and recommendations (No. 95, 1952; 165, 1981; 191, 2000) which seeks to broaden the scope and entitlement of maternity protection at work [21]. It is important to build national consensus through research and advocacy to support women in balancing a formal job and academic responsibilities with optimum breastfeeding practices when required.

Over the past years, international development scholars have used the concept of empowerment as a guiding framework in championing women's development [14, 22–24]. Some of these scholars [24] believe that empowerment is synonymous to accessing resources (economic, human and social) confer on individual’s and group’s ability (referred to as agency) to define and achieve their life’s goals or the capacity to dominate the abilities of other individuals and groups through the exercise of authority or coercion. Resources together with an agency are the instruments of individual and group potentials for making choices in life (referred to as achievement). However, access to resources depends on rules and norms governing distribution and exchange in social institutions [14].

Based on the adopted concept of empowerment, [14] the authors argued that without adequate institutional arrangements and norms that are supportive of childcare in the formal labor market and higher institutions of learning, many women of childbearing age may be denied access to higher education and opportunities to participate in the formal labor market. Based on this perspective, the concept of empowerment, as emphasized by World Bank, is very significant for women carrier progression and family life [25]. These can be seen as transcending beyond mere economic betterment of women to comprehensive well-being [26]. These concepts are strategic gender issues relating to breastfeeding as discussed in this paper. The challenges women encounter to breast feed particularly at the work place [27] and in academic settings [28] are very enormous and sometimes compels women to compromise childcare with meeting the demands of work and school.

Exclusive breastfeeding in Ghana usually declines by 52% during the third month of infants’ life; [29] a situation which is attributed to the practice of women returning to work after maternity leave at which time the infant will be three months old [30]. Studies have shown that the multiple roles of women conflict with their ability to perform optimum breastfeeding responsibilities, especially in cases where remunerated work is crucial to supplement the family budget [31, 32]. Further, the maternity protection provision of the Labor Act in Ghana does not extend to female students of higher institutions of learning who become pregnant and deliver in the course of study. This is because at best students who deliver during the course of their education can defer the course on health grounds and not just for the fact that a baby is born. Childbirth or normal delivery is not an explicit justification to differ a course in public Universities in Ghana.

Even though some studies have reported the breast-feeding challenges faced by employee mothers [33] and student mothers [34, 35] on university campuses, there is limited evidence of actions taken by university authorities to provide support for students and employee mothers (i.e., paid maternity leave for working mothers and provision for student mothers to differ a program after childbirth if required). Additionally, apart from flexibilities in allowing nursing mothers on the various university campuses to breastfeed, there is no further support given to working and/or student mothers within the general framework of promoting the development and rights of women to equal opportunities in society. The studies that have significantly explored this domain have mostly focused on breastfeeding among mothers in the informal labor market and breastfeeding related issues at the expense of those in the formal labor market [36–38]. Upon this backdrop, the question is: what support do employers and academic institutions provide for women to effectively combine their formal job or academic responsibilities with their day-to-day duties of providing optimum breastfeeding practice and childcare? Reponses to these questions will providing valuable information to chart a path forward that will inform policy and programme decisions in Ghana as well as contribute to the literature in this area.

## Methods

### Study Design

The authors adopted an exploratory-descriptive case study design with a qualitative approach for data collection and analysis. (i.e., Exploratory because the study was conducted when the authors have just begun an investigation and wished to understand the topic generally; Descriptive because the authors aimed to describe the situation at hand and case study because the study was an in-depth examination of the research topic within a real-world context of three academic institution in Ghana). The choice of this integrated design aligns with the study objective to examine the policies and programs available in public universities to support breastfeeding and childcare practices for employee and student mothers. The Human resource policies and students’ handbooks of the various Universities studied were also reviewed to examine the provisions made therein for breastfeeding on campus. The COREQ checklist [39] provided additional guidance for the study design.

### Setting

Three renowned public universities in Ghana [(University of Cape Coast (UCC), University of Education, Winneba (UEW), and Kwame Nkrumah University of Science and Technology (KNUST)] were involved in the study. KNUST is in Kumasi and covers seven square miles in the area. Sixty percent of the student population resides in private hostels due to limited on-campus hostel facilities. It has decentralized collegiate administration with six colleges since January 2005. The colleges are semi-autonomous and headed by a provost, with Deans and Directors heading the various centres, faculties, and institutes in each college [40]. UCC is located in the central region of Ghana with a current student population of 67,938. The University began operations as a College in October 1962 and has grown over the years into five Colleges with six Faculties and eight Schools headed by Provosts and Deans respectively [41]. UEW was established in 1992 with a mandate to train educationists. The University is divided into satellite campuses across Ghana and has a current student population of 59,916. Administratively, UEW operates a centralized system headed by a Vice-Chancellor [42]. These three universities have other modules of admissions (matured student admission, sandwich, weekend, and evening school admissions) in addition to the regular admissions. Such modules of admissions attract large numbers of working mothers and adults who may have reproductive intentions.

As per the maternity leave records of the participating universities, it is evident that on the average, more than ten (10) female university employees of various employment status become nursing mothers each year. Whereas the records of student mothers in this category is hard to find due to the fact that students do not usually require maternity leave in the university environment to necessitate such documentation, anecdotal evidence on the various university campuses have shown that about 20–30 regular students at various levels in their programmes become nursing mothers each year and this figures usually doubled among students offering sandwich programmes. It is very common to see such students around campus breastfeeding in the opening or sometimes even in lecture rooms as teaching goes on at the full glare of others.

### Target population

The target population for this study comprised breastfeeding employee and student mothers as well as the management of the three public universities in Ghana. The respondents for the interviews were purposively selected, whilst the participating institutions (KNUST, UCC, and UEW) were randomly selected from the other traditionally known public universities in Ghana, that were established before the year 2015 (i.e., University of Ghana, Legon; University of Development Studies, Tamale; University of Professional Studies, Accra and University of Health and Allied Sciences, Ho). The year 2015 was set as cut off point for inclusion because the year the marked the end of the United Nation’s Millennium Development Goals and provides a basis for comparison as the authors assumed that these Universities might have had relevant policies on the gender roles of women including breastfeeding policies to support student mothers as part of some strategic decisions for achieving the Millennium Development Goals in Ghana. The selection of the three universities were by simple random selection as there were other public university in Ghana from which the three were selected. Selection of participants for the interviews were however purposive because the characteristics of respondents were well defined for the purposes relevant to the study.

### Inclusion and exclusion criteria

Participants in the study were university staffs and students who at the time of data collection were breastfeeding babies less than six months of age, as well as senior staff members from key divisions of the three public universities whose activities have a direct bearing on the research topic (i.e., Human Resource Division, Quality Assurance Division, Academic Planning Division, Office of the Dean of students and the University Teachers Association (Local Branch). People who do not have these credentials were excluded from the study.

### Data collection

Data were collected by both authors between April and July 2018 among purposively selected university employees and student mothers via audio recordings, field notes, desktop review of documents including review of student handbooks and policy documents. An unstructured interview guide developed by the authors for this study was also used for data collection. The interview guide was adapted from the ILO’s maternity resource package [21], which spells out conditions for embarking on paid maternity leave. Additionally, an interview guide developed for a previous study [33] was also used as a guide. Interviews commenced after formal approvals were obtained from the three universities and respondents. The interviews lasted 40 to 60 min and discussions continued until saturation was reached when no new related issue and views no longer came up. All interviews were conducted in English. The Institutional Review Board (IRB) for research Ethics at the University of Cape-Coast gave ethical approval for the study.

### Data analysis

Data Analysis was manually done thematically by both authors. With this approach of data analysis, the data collected were transcribed immediately after each interview and analyzed using a thematic analysis approach. The transcribed data was tabulated with two columns (A and B). Column ‘A’ contained the transcribed data and column ‘B’ the field notes. This rough tabulation was done to compare the transcribed data and field notes so as to ensure that no relevant information was omitted. The synchronization of the transcribed audio and field notes constituted the final results from the field work which were presented verbatim or paraphrased as results from the interviews. Information obtained from the desk review of documents also threw more light on key issues of interest to the study objectives which complimented the field data to support the findings and enriched the discussions.

## Results

Thirty-six (36) respondents from the respective universities participated in the study. The distribution includes the Human Resource Division (6), Quality Assurance Division (6), Academic Planning Division (6), the office of the Dean of students (3), three respondents from the University Teachers Association (Local Branches) and 15 student mothers; five from each of the selected universities.

Three main themes (breastfeeding policy and programs, institutional support, and views on Breastfeeding/Childcare Support) emerged during data analysis. The breastfeeding policy and program had 3 sub-themes; institutional support on the other hand has 1 sub-theme and views on breastfeeding/childcare support have 2 sub-themes (Table 1).

**Table 1:** Main and Sub-themes

Main themes	Sub-themes
1.Availability of breastfeeding policy and programs	i.Official policies and programs of the universities
	ii.Conditions of Work (CW)
	iii.Maternity Protection Privileges
2.Institutional support for breastfeeding/Childcare	i.Childcare related Support
3.Views on breastfeeding/childcare support	i.Management Perception of Support
	ii.Perceived institutional challenges

## Availability of breastfeeding policy and programs

This category focused on existing official policies and programs of the universities that outline their position on breastfeeding and childcare among employee and student mothers. Conditions of work, related documents and maternity protection privileges are sub-themes that emerged from the main category.

i. ***Official policies and programs of the universities***

This sub-theme exhibited official policies and programs of the university on breastfeeding and childcare related supports. It is concerned with the various policy and programs provided by the universities to help student and employee mothers cope with breastfeeding and childcare. It was observed that, although the various universities had some flexible and informal arrangements to support breastfeeding mothers (i.e., taking short breaks to breastfeed, closing earlier than official working hours, and in some cases bringing nannies around the work/school environment to prompt mothers when their babies require breastfeeding). There were however no official policies and programs binding these flexibilities. A respondent explained that:

*‘We are humans, for that matter, we ensure some flexibility in these issues to ensure our nursing mothers don’t unnecessarily deprive their babies of breastfeeding when required’ (Participant 1, University 1).*

ii. ***Conditions of Work (CW)***

Despite comprehensive policies on the conditions of work with respect to childbirth in the various universities involved in this study, there were no explicit formal policy documents for breastfeeding and childcare in the three participating universities. The closest available document to childcare policy was the condition of work (CW) of the three universities which guarantee maternity protection for employee mothers in line with the Labour Laws of Ghana. The content of this document is explained to employees during orientations and copies are made available in faculties and department offices, online, and in libraries of the universities.

In addition to the CW of the universities, two of the universities had provisions in their strategic plans to provide childcare related support to both students and employee mothers. Even though one of the universities has a directorate that champions gender issues, specific policies, and programs to support childcare and breastfeeding were not available. Heads of some key units in two of the universities indicated that the strategic plan of their university has strategic goals towards promoting infants and child care at the university. One university had plans to provide on-site crèche for both student and employee mothers. An officer from the planning unit in one of the universities outlined some provisions in their strategic plan as follows.

*‘In this University’s strategic plan decisions have been made to provide a crèche...we have allocated a place and we are going to build’. (Participant 1, University 3)*

*‘This issue has never crossed our mind as an institution, but it’s worth considering for our future plans since it’s a relevant issue for our females’. (Participant 2, University 3)*

iii. ***Maternity Protection Privileges***

All officials interviewed in the three universities reported that ‘employee mothers are entitled to the statutory maternity leave but no such provision for student mothers. Some officers indicated that:

*‘Where a mother encounter complication, the case then moves beyond pregnancy the employee will have to follow the procedure for that once this is done, we extend the maternity leave’. (Participant 3, university 3).*

*‘We have provisions for paid maternity leaves for our employee mothers but there is nothing like maternity leave for student mothers. At best a student mother is allowed to defer the course either on health grounds or upon a reasonable request to nurse a newborn baby.’ (Participant 1, University 2).*

Additionally, employee mothers were found to have the privilege of taking casual leave or leave of absence where necessary. It was also noted that employee mothers are allowed to arrange for their annual leave at the expiration of their maternity leave. On resumption from maternity leave, mothers are offered the opportunity to work five days weekly or 20-h per week (5/20) for nine consecutive months instead of the usual five-days, 40-h work per week (5/40).

A review of student handbooks of the universities revealed the existence of procedures that partially support academic work and childcare. The universities provide counseling services for academic, social, and personal issues affecting students. They also have in place procedures for deferment of programs and examinations. However, deferment of programs has some limitations. As part of the policy, a student cannot defer his/her program in the first year of admission. Where applicable, deferment is limited to two semesters. Only one university had a maximum period of four semesters on grounds that the deferment period would not exceed the maximum period allowed for completion of the program.

In terms of housing or on-campus accommodation policies, none of the universities had specific accommodation arrangements for nursing mothers. This was explained that pregnancy among students is usually not predictable for the university to invest in such a policy/program.

*‘We are concern with academic work and not childbearing issues. A woman who opts to be pregnant in school is expected to make her own arrangements to nurse her baby.’ (Participant 2, University 1).*

*‘The University has plans to build mother and child hostel with facilities convenient to accommodate nursing student and employee mothers in future but not now’ (Participant 4, University 3).*

## Institutional breastfeeding support

The key barriers to breastfeeding faced by student-mothers include lack of legal protection, lack of breastfeeding-friendly university policies, inadequate availability of breastfeeding facilities, and insufficient awareness of the importance of breastfeeding among mothers. Consequently, all three universities used in the study did not have designated facilities for breastfeeding. In one of the universities, officials indicated that by convention, employee mothers are not expected to come to work with babies. The university environment is considered not conducive for nursing babies and breastfeeding break is not practiced. Some respondents indicated that:

*‘Employees are not allowed to be at work with their babies. Though this is not a policy. I think the work environment could be hazardous to the child’. (Participant 2, University 2)*

*‘No, we don’t have breastfeeding facilities or a lactation room. There is a kitchenette but since employees are not supposed to come to work with babies, I don’t think the kitchenette will serve that purpose’. (Participant 5, university 3)*

*‘It would have been very helpful if we have facilities dedicated for breastfeeding on campus so that at least our nannies can stay with our babies whilst in school so we come over to breast periodically’ (participant 6 university 3)*

In another university, an official said that the university does not regulate workplace nursing, hence have no ceiling on the number of breaks available to a mother in a day. Despite this flexible work environment aimed to enhance child care, most employee mothers choose not to come to work with babies so as to avoid interfering breastfeeding with their work.

*‘Breastfeeding staff can break anytime to breastfeed their babies provided there is no pressure or workload on them’. (Participant 2, University 1).*

Likewise, there is no policy in any of the Universities that prevents student mothers from nursing their babies on campus.

*‘Although this is an academic environment, people have their rights to childbirth and childcare hence, the university does not prevent student and employee mothers from nursing their babies in any of these university campuses. What we discourage however is for babies to be left to loiter around unattended to and disturbing official work and lectures as usually seen during our sandwich and weekend programs’(Participant 3, university 2).*

Only one of the universities has a purposefully built but privately managed on-site crèche situated in close proximity to lecture halls and administrative offices. The remaining two universities didn’t have on-site crèche for employees and student mothers.

*‘We a crèche close to our lecture hall where we send our babies and come over to breast feed them periodically’. (Participant 3, University 1)*

- ***i. Childcare related support***

The practice of five-day 20-h work per week (5/20) “half-day” was found to be substitutive to breastfeeding break. As part of flexible work schedule practices, employee mothers can arrange with their immediate boss on which time of the day to report to and close from work for their 4-h work per day. A human resource officer explained further:

*‘There is no breastfeeding break because of the half-day. But there is flexibility, for instance, if a staff is supposed to report at 8 am and close at 12 pm, she can arrange with her head so she can come at 9 am and close at 1 pm’. (Participant 7, university 3)*

Officials interviewed indicated that they resort to the various internal arrangement because the universities have no standard procedures on flexible work schedules. In one of the universities, an official mentioned that the Vice-Chancellor is supportive of women’s welfare and that housing arrangements for students are made to favor female students. There were however no future plans for on-campus accommodation for student mothers*.* Rather, the universities have breastfeeding promotion and education programs for employee mothers at the universities' hospitals as part of maternity services.

*‘At the university hospital, we have a complete area for pregnant women...... the staff is aware of it and they go there for antenatal, weighing and receive breastfeeding education’. (Participant 4, University 1)*

*‘I don’t think we have such programs, but we have breastfeeding awareness creation in the departments annually’. (Participant 4, University 2)*

## Views on breastfeeding/childcare support

This category provides information on university management perception about the workplace and on-campus support for breastfeeding and childcare. Management perception of support and perceived challenges are the sub-categories.

i. ***Management Perception of Support***

In one of the universities, it was mentioned that management considers breastfeeding as an important element of infant care due to its immense benefit to mothers and infants. An official from another university said providing support for breastfeeding and childcare is about the welfare of employees and their children, as such the university will support it. Another official also mention some health benefits of breastfeeding and suggested that it is important to support employee mothers. However, no mention was made of support for student mothers to help them combined childcare and academic work effectively.

ii. ***Perceived institutional challenges***

Financial cost emerged as a major challenge hindering universities' support for breastfeeding and childcare. All officials interviewed concluded that maternity leave is expensive in terms of loss of man-hours. Cost of relieving duties, difficulties in finding a replacement for staff going on maternity leave, and/or work overload on existing staff. Increased work overload often results in fatigue and low productivity among staff.

*‘Someone may have to take on extra responsibility and the person may suffer stress, sometimes we have vacant positions due to maternity leaves. (Participant 8 University 3)*

*‘The proposed six months maternity leave would have been the best.so that mothers can have ample time to breastfeed their babies, but the vacancy it will create would be difficult to fill. It also comes with extra cost anyway’. (Participant 4 University 2)*

Human resource representatives interviewed suggested that future review of the maternity protection provision of the Labor Act should consider extending the period of the “half-day” currently enjoyed by mothers instead of the maternity leave duration. They also proposed the implementation of baby and child-friendly work environment initiatives such as the provision of breastfeeding rooms and on-site crèche in place of extension of the maternity leave period. Initiatives such as allowing employee mothers take maternity leave in addition to current and ensuing year’s annual leave were proposed.

## Discussion

The findings suggest gaps in breastfeeding and childcare provisions in public universities in Ghana, which have a policy and program implications as the present arrangement is discriminatory and nurtures gender inequality. Literature on women empowerment within the Arab world, have indicated that advocating for women’s right to work in the formal labor market, acquire property, and freedom to speak on issues relating to their safety and sexuality goes beyond providing access to micro-credit and sewing machines [42]. The concept of women's empowerment however focuses on the capability of women for self-determination by taking control over their own circumstances and to realize their aspirations in order to live a life they have reason to value [14]. These includes the ability of women to define their goals, have meaningful choices, and to act to achieve desired outcomes. This framework implies that efforts to promote women’s empowerment and rights to equal opportunities, especially in the Ghanaian context, also need to transcend mere livelihood empowerment and social assistance programs to genuine initiatives that weaken social and institutional barriers to equality.

Various policy concerns and questions have been posed as to how social protection can provide social justice for women [43]. With respect to the cultural context of Ghana, it has been observed that ‘if young people are given voice to express how work, education, social networks, and culturally-bound notions of responsibility are linked and how they perceive the opportunities and constraints on their ‘life chances’ they will better manage their lives as they develop into responsible adults [2]. There are various cultural believes, norms and practices including whether women should have limited or no education that impedes women carrier development in some Africa countries [44, 45].

Official breastfeeding and/or childcare policies for employees and student mothers were not available in all the universities. Documents analyzed showed that the labor Act does not fully address the provisions of convention 183 of the ILO (Maternity protection at work, p:3). This shortfall is reflected in the conditions of work (CW) of the three public universities. The CW of the universities provides 12-weeks maternity leave. One commendable provision in the CW is the five-day 20-h work per week (5/20) offered to mothers returning to work from maternity leave. The anecdotal evidence indicating absence of comprehensive official breastfeeding and childcare policies in the public universities in Ghana corroborate national survey findings of colleges and universities even in developed countries such as in the United States of America where empirical evidence has shown that only 5 out of 139 institutions had a formal policy on lactation [35]. This finding is an indication that unavailability of comprehensive breastfeeding and childcare policies in institutions of higher education is a challenge globally despite the availability of various international policy and advocacy drives to this effect [46]. These are indications that much more is required in implementing these policies to ensure optimal childcare by nursing mothers at the work place including in academic institutions. When these policies are adequately implemented, new mothers returning to school after childbirth will not face barriers within academic settings to meeting their goals for exclusive breastfeeding.

The ILO convention (1981) and the National Labour Commission of Ghana, stresses the need to provide family-friendly working hours and childcare services with suitable facilities for workers to cope with childcare needs [47]. Yet the universities have no breastfeeding rooms for employees and student mothers. Breastfeeding break was not consistent among universities studied since the various universities had different informal breastfeeding polices. Even though officials provided some reasons for not allowing it, such as the unconducive nature of the office environment for breastfeeding and childcare, partial implementation of breastfeeding break by the universities was mainly due to the non-availability of breastfeeding rooms and childcare facilities to provide mothers time and privacy to breastfeed. Although some previous studies.

[33, 48] have documented the importance of space at workplaces for breastfeeding and childcare, a key observation from the current study shows that there are many bureaucratic bottle necks for implementing these good policies in the universities involved in this study. For instance, there are various stakeholder consultations, collaborations, and funding support to be done to move this idea, which might not be a top priority for most of the universities in the short term. These findings are in tangent with a similar previous study [49] which reported that working nursing mothers face many challenges in combining work requirements and breastfeeding demands at the workplace.

Flexible work arrangement is not standardized across units in the universities and its left to the discretions of the various heads of departments and units. This has the potential to deny employee mothers the flexibility required to combine work and optimum breastfeeding and childcare in circumstances where the predisposition of ahead of unit or department is not supportive of nursing and childcare. Only one out of the three universities studied was found as an exemplar in childcare arrangements. This university has a purposefully built crèche. However, the location of the facility is quite distant from some departments; a situation that challenges mothers’ ability to provide optimum breastfeeding.

In line with Kabeer’s concept of empowerment [14], it is the authors expectation that, women associations in the various universities would be encouraged to advocate and lobby various stakeholders for support to establish breast feeding facilities on campus just as most public universities in Ghana have public–private collaborations for developing hostel facilities on university campuses. Similar arrangements could be made to set up creches that will be evenly distributed in the various campuses in the universities to meet the needs of both students and workers who are nursing mothers in the various universities. The prospects of this could results in good child health and sound maternal mind for studies and/or work once there is an assurance of child safety and proximity to breastfeed on demand. This approach will be very cost effective in terms of the university recouping its investment in establishing and commercializing this facility. Man, hours lost coming to work/lectures late and to leave work/lectures early in order to cater for a child would have been saved to ensure productivity.

It must be noted that the maternity protection provision of the labor act and by extension, CW of the universities do not extend to student mothers. The only privilege available to students and/or expectant mothers in the course of their study program is the option for one-year deferral, in cases of pregnancy or childbirth. Yet, the deferment policies of public universities are subjected to certain limitations and exclude first and final year students from enjoying such privileges. Therefore, students who are not sure of readmission prefer combining these two incompatible roles [33]. This observation is an indication that there are very separate maternal and childbirth privileges for students and workers in the various universities in Ghana.

Although the study did not assess university management knowledge on the benefit, they stand to gain from providing breastfeeding support at the workplace, it was evident that they are aware of these benefits. Yet, no remarks were made regarding breastfeeding and childcare support for student mothers. Officials rather pointed out likely challenges such as Cost and work overload to come their way should there be an extension of maternity leave beyond the current period. It was also noted that making provisions to support student mothers to provide the necessary care for their babies whilst pursuing academic programs on the various university campuses is not one of the immediate priorities of these universities. Although it was observed that such provision is desirable, it is not mandatory nor an urgent need considering the population of students faced with such challenges.

The observation that Universities in the study do not have adequate policies, programs, and facilities to support breastfeeding and childcare for employee and student mothers, to a large extent discriminate against women’s right to equal opportunities for work, education and their ability to freely exhibit their fundament human rights of childbirth by choice in an academic environment. The absence of a formal breastfeeding/childcare policy and program in the various universities could have negative implications for achieving the Sustainable Development Goals 3 and 5 in Ghana as it can create avenues for marketing of breast-milk substitutes and patronage due to inability of mother to breastfeed babies on demand [50]. This deficiency has the potential to affect optimal child nutrition, growth and development as well as negatively affecting women’s rights to equal opportunities for work and higher education in Ghana by nursing children to grow before embarking on any form of formal employment and education at the tertiary level.

### Limitations

The study design has a limitation in scope because it is qualitative and using a small sample size of breastfeeding mothers from which findings cannot be generalized across all public universities in Ghana. The study was also silent on work performance appraisal reports and academic performance of employees and student mothers to critically establish the effects of on-campus childcare on work and academic performance of participants for the required interventions.

## Conclusion

The authors in this paper assessed the availability and implementation of breastfeeding policies and programs in selected public universities in Ghana. It was evident from the study results that there is a lack of formal breastfeeding policy/program in the various universities that were studied. These observations could have negative implications for achieving Sustainable Development Goals 3 and 5 in Ghana. The authors, therefore recommend that the national accreditation board for tertiary institutions should consider the possibility of incorporating childcare modalities as part of the requirements for the accreditation of universities in Ghana to enforce the law. This when done, will enhance on-campus facilities for breastfeeding and childcare which will empower more women of reproductive age to combine their reproductive intentions with academic pursuits at the tertiary level. Additionally, a national qualitative survey is also recommended to assess the current status of breastfeeding and childcare support from the perspectives of employers, employees, and students in the various tertiary institutions in Ghana to guide policy and program decisions/interventions.

## Data Availability

Data for the study can be obtained from the corresponding author upon reasonable request.

## Abbreviations

CW: Condition of work
GLSS: Ghana Living Standard Survey
HR: Human Resource
ILO: International Labor Organization
KNUST: Kwame Nkrumah University of Science and Technology
COREQ: Consolidated criteria for reporting qualitative research
UN: United Nations
UEW: University of Education, Winneba
UNESCO: United Nations Educational, Scientific and Cultural Organization
UCC: University of Cape Coast
UTAG: University Teachers’ Association of Ghana
